# A PMAxx^TM^ qPCR Assay Reveals That Dietary Administration of the Microalgae *Tetraselmis chuii* Does Not Affect *Salmonella* Infantis Caecal Content in Early-Treated Broiler Chickens

**DOI:** 10.3390/vetsci9090487

**Published:** 2022-09-08

**Authors:** Joselyn Corrales-Martinez, David Ortega-Paredes, Miroslava Anna Šefcová, César Marcelo Larrea-Álvarez, Sofía de Janon, José Medina-Santana, Gabriel Molina-Cuasapaz, Christian Vinueza-Burgos, Viera Revajová, Marco Larrea-Álvarez, William Calero-Cáceres

**Affiliations:** 1 Bacteriophage Research Association, Ambato 180103, Ecuador; 2 Research Unit, Life Science Initiative (LSI), Quito 170102, Ecuador; 3 Facultad de Ciencias Médicas Enrique Ortega Moreira, Carrera de Medicina, Universidad Espíritu Santo, Samborondón 092301, Ecuador; 4 Unidad de Investigación en Enfermedades Transmitidas por Alimentos y Resistencia a los Antimicrobianos (UNIETAR), Facultad de Medicina Veterinaria y Zootecnia, Central University of Ecuador, Quito 170129, Ecuador; 5 Department of Morphological Disciplines, University of Veterinary Medicine and Pharmacy, 040 01 Košice, Slovakia; 6 UTA RAM One Health, Department of Food and Biotechnology Science and Engineering, Universidad Técnica de Ambato, Ambato 180103, Ecuador

**Keywords:** PMAxx^TM^-based qPCR, *Salmonella enterica* subsp. *enterica* serovar Infantis, bacterial viability, caecal content, broiler chickens, *Tetraselmis chuii*

## Abstract

**Simple Summary:**

*Salmonella enterica* subsp. *enterica* serovar Infantis (*S.* Infantis) has emerged as a relevant serovar commonly reported in poultry products, which represents a potential public threat. Microalgae synthesize molecules that exert positive effects both on chicken performance and health. *Tetraselmis chuii* produces fermentable polysaccharides capable of influencing caecal diversity. We tested if the administration of *T. chuii* could alter caecal microbiota in favor of a reduced *S.* Infantis load. Animals were fed a microalgae-based diet and challenged with bacteria on day 4. Two days after infection, caecal samples were taken and the viable load of *S.* Infantis was estimated utilizing a PMAxx^TM^-based qPCR method, which was developed and tested for assessing the differences between groups. Dietary inclusion of the chlorophyte did not alter bacterial viable load; however, the method proved to be efficient, sensitive, and repeatable. Certainly, the approach used herein could be applied in studies evaluating the effects of different treatments on *Salmonella* caecal colonization.

**Abstract:**

*Salmonella enterica* serovars cause infections in humans. *S. enterica* subsp. *enterica* serovar Infantis is considered relevant and is commonly reported in poultry products. Evaluating innovative approaches for resisting colonization in animals could contribute to the goal of reducing potential human infections. Microalgae represent a source of molecules associated with performance and health improvement in chickens. *Tetraselmis chuii* synthesizes fermentable polysaccharides as part of their cell wall content; these sugars are known for influencing caecal bacterial diversity. We hypothesized if its dietary administration could exert a positive effect on caecal microbiota in favor of a reduced *S.* Infantis load. A total of 72 one-day-old broiler chickens (COBB 500) were randomly allocated into three groups: a control, a group infected with bacteria (day 4), and a group challenged with *S.* Infantis but fed a microalgae-based diet. Caecal samples (*n* = 8) were collected two days post-infection. A PMAxx^TM^-based qPCR approach was developed to assess differences regarding bacterial viable load between groups. The inclusion of the microalga did not modify *S.* Infantis content, although the assay proved to be efficient, sensitive, and repeatable. The utilized scheme could serve as a foundation for developing novel PCR-based methodologies for estimating *Salmonella* colonization.

## 1. Introduction

*Salmonella enterica* is one of the main causes of foodborne disease, representing a worldwide problem for public health systems [1]. This species consists of more than 2600 serovars described to date [2,3]. *S. enterica* serovars can cause infections in humans ranging from gastroenteritis, associated with non-typhoidal *Salmonella* (NTS), to typhoid fever [4]. NTS are estimated to cause more than 93.8 million infections and 155,000 deaths per year globally [5,6]. The main serovars of NTS related to human infections are *S*. Typhimurium and *S*. Enteritidis. However, *S. enterica* subsp. *enterica* serovar Infantis (*S.* Infantis) has positioned itself among the five most relevant serovars reported in poultry products [3,7,8,9,10]. The spread of this serotype represents a potential threat to public health since it is found in about 90% of the total number of isolates in chickens [11]. Additionally, some isolates have shown resistance to third-generation cephalosporins and other antibiotics including fosfomycin, aminoglycosides, sulfonamides, and chloramphenicol [12]. These characteristics have been associated with a plasmid known as PSI (plasmid of *Salmonella* Infantis). It has also been reported that resistance integrons could be playing an important role in the mobility of these traits [13]. Therefore, *S.* Infantis isolates harboring resistance factors can be considered potential etiological agents of intestinal and extra-intestinal human infections associated with chicken products [12].

Recently, research has focused on testing novel strategies for diminishing *Salmonella* colonization in chickens, which appears imperative for reducing the incidence of potential human infections [14]. For instance, probiotics have not only proved convenient for reducing pathogenic *Salmonella* [15,16], but also for ameliorating intestinal health [17,18,19,20,21]. Other microorganisms, such as microalgae, have also shown beneficial effects with regard to animal performance, product quality, and health [22]. In particular, dietary administration of *Tetraselmis chuii* ameliorated intestinal architecture and induced goblet cell differentiation in broiler chickens [23]. Moreover, this species, as well as other species of *Tetraselmis*, synthesizes fermentable polysaccharides as part of their cell wall content [24,25]. These molecules are known for stimulating the activity and growth of probiotics in the digestive system of animals and are thus considered capable of modulating gut microbiota [26]. In laying hens, for example, administration of a macroalgae-enriched feed (*Chondrus crispus*) showed reduced levels of *S.* Enteritidis in caecal content compared to control conditions [27]; this inhibitory effect has been associated with the population of probiotics induced by the presence of seaweed-derived polysaccharides [28].

In this study, we aimed at testing if the administration of the chlorophyte *T. chuii* could exert a positive effect on caecal microbiota in favor of a reduced *S.* Infantis load. For this purpose, we designed and evaluated a method based on a PMAxx^TM^ qPCR assay for a rapid estimation of viable *S.* Infantis load.

## 2. Materials and Methods

The experiments were conducted following the guidelines for poultry management provided by the Agency for the Regulation and Control of Phytosanitary and Animal Health (AGROCALIDAD, technical resolution n◦ 0017). The Ethics Committee on the Use of Animals in Research and Teaching of the San Francisco de Quito University (USFQ) revised and approved the related protocols (reference number: 2020-008). The study was carried out in the Experimental Centre for Animal Research of the Veterinary Medicine Faculty, Central University, Ecuador. The facility is situated 23 km southeast of Quito, in the parish of Uyumbicho.

### 2.1. Microalgae Biomass

The freeze-dried powder of *T. chuii* was obtained from Necton S.A., Olhão, Portugal. This biomass possesses crude protein (35%), crude fat (5%), and crude ash (30%) (https://necton.pt/, accessed on 2 June 2021). The experimental diet was provided with a dose of 20 g/kg of feed (2%). *T. chuii* belongs to the phylum Chlorophyta, which is associated with the synthesis of chlorophylls (a and b) as well as some carotenoids including violaxanthin, antheraxanthin, zeaxanthin, neoxanthin, and lutein [29].

### 2.2. Bacterial Preparation 

Pure cultures of *S.* Infantis (1 × 10^9^ CFU/mL) were used; recovery was performed on Xylose, Lysine, and Deoxycholate (XLD) differential selective medium at 37 °C for 24 h. Then, a characteristic colony was selected for biomass generation in buffered-peptone water (as liquid culture) and incubated at 37 °C for 18 to 24 h with constant agitation. The biomass recovered from the liquid culture was placed in Falcon tubes and centrifuged at 500× *g* for 45 min to concentrate the biomass. Finally, the pellet was resuspended using a saline solution (NaCl 5%) until obtaining an OD_600_ of approximately 1.0. The solution was numbered in series in plate count agar, and adjusted approximately at 1–2 × 10^7^ CFU/0.1 mL.

### 2.3. Determination of S. Infantis Load

Studies reporting the use of probiotics and other alternatives for modulating *S.* Infantis caecal content have predominantly used culture-based approaches [15,30]. Indeed, that is the conventional method for *Salmonella* detection, although it is considered laborious and requires from three to seven days for results. Moreover, the sample must undergo a process of enrichment [31]. This could create a bias when estimating the effects of treatments on bacterial viable load. Thus, molecular approaches appear advantageous. PCR, nonetheless, is not recommended due to its incapacity to discriminate between death, damaged, and viable cells [32]. Hence, we aimed at testing a methodology using the bacterial viability dye PMAxx^TM^ that covalently binds to the DNA of dead cells obstructing their amplification by PCR; this technique has proved useful for the selective detection of viable cells [33,34,35]. Assessments were carried out using the genomic DNA of *S.* Infantis, and the analytical performance of the assay (i.e., specificity, amplification efficiency, and reproducibility) was determined.

#### 2.3.1. Cultivation of *S.* Infantis and Extraction of Genomic DNA

The strain U1068s, obtained from the Foodborne Diseases and Antimicrobial Resistance Research Unit (UNIETAR), was used. Its complete genome, with a size of 5,003,989 bp, is deposited in the NCBI database; accession number DAEUGI00000000000000.1. The genome was screened for the *invA* gene using Geneious Prime [36]. Bacterial biomass was generated, as described in Section 2.2, on three different days (*n* = 3). DNA extraction was performed independently from these cultures using the Spin Column Soil DNA Miniprep kit (Biobasic, Markham, Canada) according to commercial guidelines. Extracted DNA was concentrated using a Savant DNA 120 speedvac concentrator (Thermo Scientific, Waltham, MA, USA) until 20 µL. The amount of DNA was estimated using a Nanodrop 2000 spectrophotometer (Thermo Scientific, Waltham, MA, USA), following the manufacturer’s instructions. Values of A260/280 equal to or greater than 1.6 were considered acceptable. DNA integrity was assessed by electrophoresis (Figure S1), and samples were stored at −20 °C until analysis.

#### 2.3.2. Amplification of the *invA* Gene

A standard was generated on an ECO real-time thermal cycler using Eco Studio™ Real-Time PCR software (Illumina, San Diego, CA, USA) from 10-fold serial dilutions of DNA from previously extracted pure cultures (15.7 ± 0.4 ng/μL, *n* = 3) (Supplementary Data). Dilutions ranged from 10^−1^ to 10^−10^ with a final volume of 450 µL. For qPCR, the primers provided by the PMA™ Bacterial Viability qPCR kit (Biotium, Fremont, CA, USA) were used. Sequences are as follows: Forward 5′-ATTCTGGTACTAATGGTGGTGATGATGATC-3′ and Reverse 5′-GCGCCAGGCTATCGCCAATAAC-3′; these allowed the amplification of the *invA* gene, a fragment considered suitable for *Salmonella* detection [37]. Primer specificity was confirmed in silico with Primer-BLAST [38]. The PCR reaction mixture was prepared by adding the components to each PCR tube, or well; negative controls were prepared with sterile dH2O (Table S1). The qPCR reaction of *S.* Infantis *invA* was run in an initial denaturation cycle at 95 °C for 5 min, followed by 40 cycles of amplification at 95 °C for 5 s (denaturation), and at 60 °C for 60 s (30 s for annealing and 30 s for extension). Fluorescence detection was performed at the end of each hybridization step, and a melting curve analysis (on a ramp from 55 to 95 °C) was included to identify dissociation peaks. The threshold was established manually at 0.099, above noise signals.

#### 2.3.3. Staining of Viable and Non-Viable Salmonella Cells Using PMAxx^TM^

**Figure 1:**
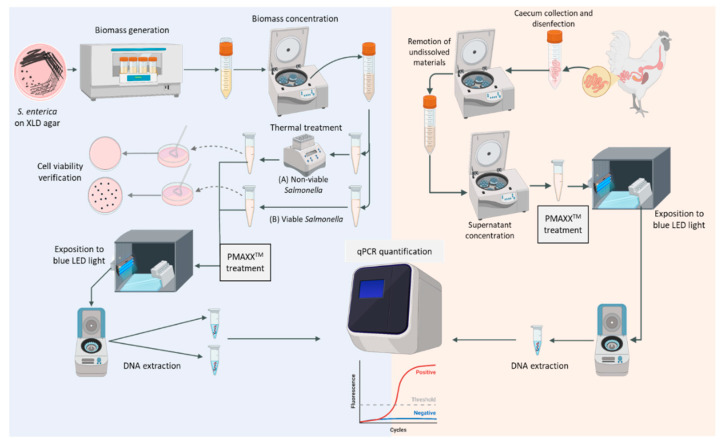
Flowchart of the *Salmonella* cultivation, thermal shock, PMAxx^TM^ treatment, cell viability verification, and qPCR estimation (left-hand side). Flowchart of caecal collection and further analyses (right-hand side). Image generated with Biorender (https://biorender.com/) (accessed on 7 June 2022-Agreement N° KH24938MG0).

*Salmonella* biomass was generated as described in Section 2.2. Two different bacterial samples (viable and non-viable) were prepared in 1.5 mL microtubes. For the non-viable culture, 1 mL of culture was exposed to 95 °C for 5 min in a dry block heater; whereas, the viable culture was not pretreated. Then, 20 mM of the PMAxx^TM^ stock (Biotium, Fremont, CA, USA) was rapidly thawed in low light to prepare a working solution by diluting it in water to 5 mM. The final dye concentration was kept at 25 µM as detailed in the guidelines [39]. For the staining of *Salmonella* cells, 6.5 µL of the PMAxx^TM^ working solution and 250 µL of PMA Enhancer reagent (Biotium, Fremont, CA, USA) were added to 1 mL of the viable and non-viable cultures. Subsequently, treated samples were incubated in the dark for 10 min and afterward exposed to blue LED light from an illuminator (Safe Imager^TM^, Invitrogen, Waltham, MA, USA) at a distance of 20 cm for 15 min. Homogenization was performed manually. Finally, the samples were centrifuged at 13,300× *g* for 5 min and the supernatant was discarded leaving the bacterial sediments for DNA extraction. Amplification of the *invA* gene was performed three times per sample as detailed in Section 2.3.2. The entire procedure was performed on three different days *(n* = 3). Bacterial cell death was verified by plating 100 μL of the non-viable sample on XLD agar and 10 µL of a 1:100 dilution of the viable sample on a different plate. Subsequently, plates were incubated overnight at 37 °C and checked for colony growth during the next 48 h. Figure 1 summarizes the pipeline followed for cultivation, PMAxx^TM^ treatment, DNA extraction, and estimation of viable load.

### 2.4. Experimental Setup

Seventy-two 1-day-old broiler cock chickens (COBB 500) were randomly divided into three experimental groups. The control group in which animals were left untreated, the Se group in which birds were infected, orally, with *S.* Infantis on day 4, and the SeTc group in which animals were also infected, but fed an enriched diet including *T. chuii* biomass during the entire experiment (6 days). Birds were fed a commercial diet (crumble) with no coccidiostats, antibiotics, or probiotics (starter, 0–8 days) (Table S2). Feed and water were provided ad libitum. Animals were allocated in pens (4 m × 2 m), each containing eight subgroups (1 m × 1 m) with three animals. One bird per subgroup was selected, thus 8 animals per pen were sampled (*n* = 8). Subgroups were considered the experimental unit as the screened animals were housed separately, so treatment conditions and experimental intervention could not influence others on the evaluated parameters [40]. Humidity was kept between 50–70%. During the first 24 h, light was provided continually (intensity 30–40 Lux); then, from day 2 light was restricted to 23 h until the end of the trials. The temperature was maintained at around 31 °C. The floor was covered with hardwood shaving. Management and housing abided by the COBB 500 Management Guide [41]. On day 6, one bird per subgroup was selected for sampling, electrically stunned, and euthanized by bleeding. Caecal sections were collected and disinfected with 96% alcohol. 1 g of caecal content (one per animal) was weighed into 50 mL tubes, and phosphate-buffered saline (PBS) (9 mL) was added. Samples were centrifuged at 900× *g* for 1 min at 4 °C to remove undissolved materials. Supernatants were transferred into fresh tubes, centrifuged at 2200× *g* for 30 min at 4 °C, and pellets were treated with PMAxx™. Subsequently, DNA was extracted using the QIAamp Fast DNA Stool Mini Kit (Qiagen, Hilden, Germany) and qPCR was performed as described above (Figure 1). Finally, the standard curve generated previously was used to quantify the DNA copy number from the cycle quantification values (Ct) and qPCR data (log_10_ DNA copy number). Results were expressed as viable *Salmonella* load per gram of caecal samples.

### 2.5. Statistical Analyses

For statistical analysis, normality and homogeneity of variance were determined using the Shapiro–Wilk’s test and Levene’s test, respectively. As data were non-normally distributed, the Kruskal-Wallis test along with the Mann-Whitney U test was used to determine differences between groups. Since the distribution was non-symmetrical, medians were used to show data as they represent more appropriately the center of distribution in these conditions. Significance was set at *p* < 0.05. Analyses were carried out in MATLAB^®^ version 9.9.9341360 (MathWorks, Natick, MA, USA) (R2016a).

## 3. Results

### 3.1. Amplification of the invA Gene

**Figure 2:**
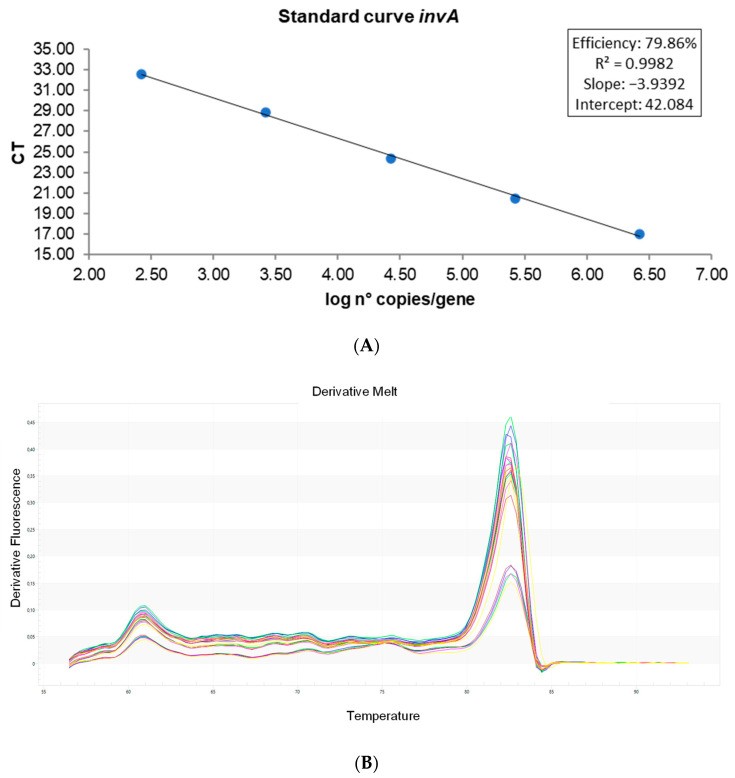
Amplification efficiency (**A**) and melting curve analysis (**B**) of primers targeting the *invA* gene. Ct values are averages from three measurements (*n* = 3).

The DNA obtained from pure *Salmonella* cultures was serially diluted for the construction of the standard curve. Per dilution, Ct values were averaged *(n* = 3) and then plotted against the logarithm of their concentrations. Figure 2A shows a linear trend with regard to dilutions, with a correlation coefficient of 0.998 and an efficiency close to 80%. Melting curve analysis demonstrated the specificity of primers, and the amplicon (287 bp) showed individual peaks with some variations in intensity. The average melting temperature (Tm) obtained for *invA* was 82.45 (Figure 2B). The equation used for quantification was: log n° copies/gene = (42.084 − Ct)/3.9392. The threshold for all the assays was established manually at 0.099, above noise signals.

**Figure 3:**
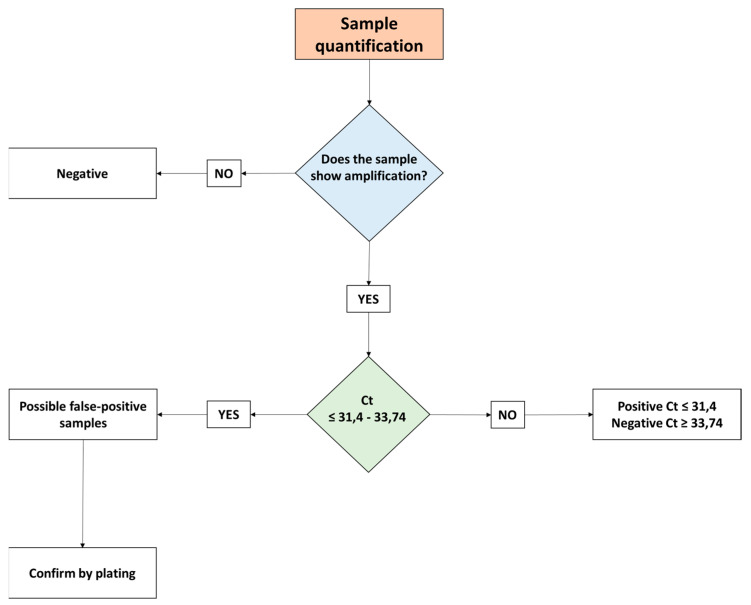
Decision diagram for quantification of samples according to the detection limit.

According to the standard curves, the experimental detection limit was set at 264.39 copies of the *invA* gene. However, modification of these limits was necessary at around 31.4 Ct, which is equivalent to 710 copies of *Salmonella*. This was related to the application of the bacterial dye treatment, as the presence of *invA* was still detected at 31.4 Ct. These unknown samples can be considered positive only up to a Ct of 31.4 and negative when having a Ct ≥ 33.74. Values between Ct 31.4 and 33.74 should be considered with caution, as they could represent false positives. Consequently, it is recommended to follow a scheme based on the aforementioned Ct values, especially for detecting potential false positives among samples (Figure 3). 

In addition, the assay showed good intra-assay repeatability. Table 1 shows the Ct values, standard deviation (SD), and correlation coefficient (CV) (Supplementary Data).

### 3.2. Inhibitory Effects of PMAxx^TM^

**Figure 4:**
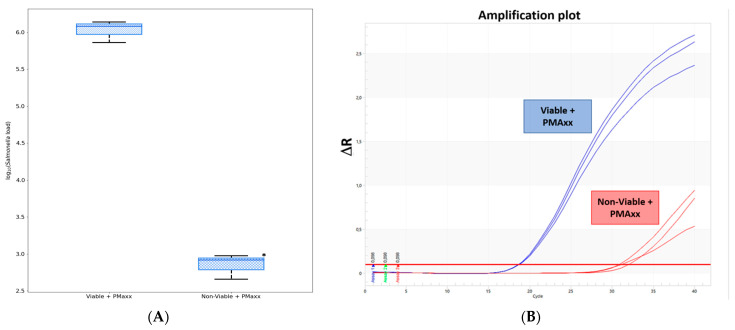
Effect of PMAxx^TM^ treatment on target DNA amplification of viable and non-viable *Salmonella* cultures. Values are medians plus their respective interquartile ranges *(n* = 3). Asterisk denotes differences between groups (**A**). Differences in Ct values (**B**).

Evaluation of the inhibitory effects of PMAxx^TM^ was performed by qPCR of DNA obtained from viable and non-viable *Salmonella* cultures. The results observed in Figure 4A show that 6.25 µL of the PMAxx^TM^ dye, at a concentration of 25 µM, inhibits DNA amplification of the dye-treated non-viable *Salmonella* culture compared to the untreated non-viable one. Furthermore, the higher *Salmonella* load of the viable group confirmed that PMAxx^TM^ penetrated only the DNA of cells with compromised cell membranes (Supplementary Data). Also, Figure 4B shows the inhibitory effect of the dye in relation to the amplification plot. A reduction of the amplification signal, of about 10 Ct, was observed in the non-viable culture treated with PMAxx^TM^.

### 3.3. Effects of Microalgae Administration on S. Infantis Caecal Load

**Figure 5:**
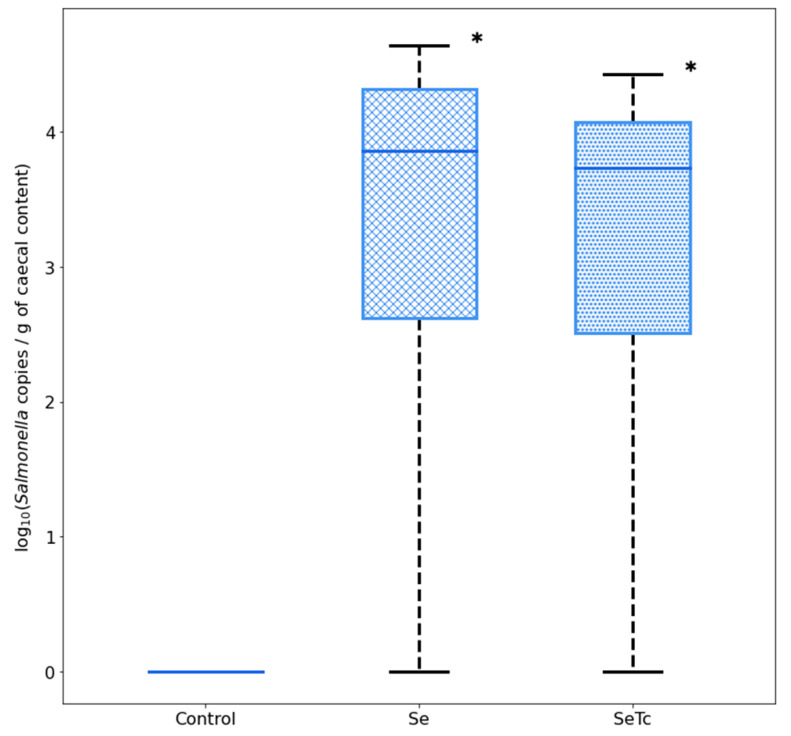
Quantification of *S.* Infantis load in samples of broiler chickens treated with a microalgae-based diet. Values are medians plus their respective interquartile ranges *(n* = 8). Asterisk denotes differences with the control group. Se: *S.* Infantis group; SeTc: *S.* Infantis + *T. chuii* group.

The assay was evaluated in broiler chickens infected with *S.* Infantis, but fed with a microalgae-based diet. Dietary administration of microalgae biomass did not influence *S.* Infantis content load, as levels were similar to those found in *S.* Infantis-challenged chickens. In control animals, no traces of *S.* Infantis were found (Figure 5) (Supplementary Data). 

## 4. Discussion

*Salmonella enterica* is one of the main causes of foodborne disease worldwide [1]. In humans, serovars of this species can not only cause typhoid fever, but also gastroenteritis associated with NTS. *S.* Infantis has arisen as a relevant serovar reported in poultry-derived products [3,7,10]. Thus, the spread of *S.* Infantis could be considered a potential public health menace [11]. Testing alternative steps to decrease *Salmonella* colonization in chickens is important for reducing the incidence of human illness [14]. However, studies assessing the effects of probiotics or prebiotics rely on the use of a culture-based methodology [15,30]. In this study, we have demonstrated that dietary inclusion of the green microalgae *T. chuii* did not alter *S.* Infantis caecal content by using a PMAxx^TM^ qPCR assay. 

Primers provided with the kit proved to work as amplicons showed discrete peaks, although amplification efficiency was close to 80%. Efficiency could be improved by using custom primers, as demonstrated in previous research [35]. DNA amplification from non-viable cells was inhibited by the presence of the dye. The amplification from the viable group, treated with the dye, confirmed that PMAxx^TM^ could not penetrate cells with uncompromised membranes. The PMA^TM^ Bacterial Viability qPCR kit (Biotium, Fremont, CA, USA) recommends a concentration of at least 25 to 50 µM [39]. In our assay, the final concentration of the dye was kept at 25 µM, which demonstrated that using a minimal concentration did not have an impact on the effectiveness of the test. 

It could be argued that the use of *T. chuii* biomass might exert a positive effect on caecal microbiota in favor of a reduced *S.* Infantis load. However, the administration of microalgae biomass did not influence *S.* Infantis caecal levels. Similarly, the utilization of *Chlorella* sp., another Chlorophyte, did not alter the content of *Salmonella* in the caecum of broiler chickens, notwithstanding the increment in the *Lactobacillus* population [42]. Probiotics, on the other hand, have been used for reducing the content of *Salmonella* in birds [16]. In particular, reduced levels of *S.* Infantis in the gut of broiler chickens and pigs have been observed after supplementation of probiotic bacteria [15,43]. These bacteria are known for reducing the growth of pathogens by stimulating the immune system, synthesizing short-chain fatty acids (SCFA), secreting antimicrobial compounds, and competitively excluding other bacteria [44]. We determined that dietary microalgae did not affect bacterial colonization during the early stages of growth; however, the utility of *T. chuii* should not be discarded as it has proved to ameliorate intestinal architecture in older animals (35-day-old chickens) [23]. Thus, further research ought to investigate the effects of this microalga on caecal microbiota, especially during the grower and finisher phases. Certainly, testing these photosynthetic microorganisms remains a significant challenge for developing dietary prebiotics suitable for resisting pathogen infection. 

Despite the negative results concerning microalgae biomass administration, the detailed approach proved useful for estimating *S.* Infantis viable load from caecal samples. Thus, it could be utilized as an easy and accessible scheme in investigations comparing the effects of diverse treatments on *Salmonella* colonization, as many of them rely on culture-based approaches [15,30]. Previous research has shown that results obtained from PMAxx ™-based qPCR assays correlate positively with data generated from culture-based methods [35,45]. In fact, this qPCR technique has demonstrated improved sensitivity [35,45]. Therefore, the present approach has the potential for being used not only in the aforementioned studies but also in the poultry farm context. Nonetheless, to attain such a goal, proper standardization and validation of the method must be performed, although the data revealed herein serve as a foundation for its development. 

## 5. Conclusions

*S.* Infantis represents a threat to public health as it is considered one of the most relevant serovars reported in poultry-derived products. Testing novel strategies to reduce bacterial colonization could contribute to the overall goal of decreasing potential human infections. Microalgae have arisen as a promising source of active biomolecules that could exert positive effects on both the performance and health of birds. Fermentable polysaccharides, in particular, are able to modulate bacterial microbiota, and *T. chuii* is known for synthesizing these sugars as part of their cell wall content. However, dietary inclusion of this microalga did not exert an influence concerning *S.* Infantis viable load in the caecum of 6-day-old broiler chickens. Undoubtedly, further research is needed to reveal if this treatment could modulate bacterial colonization at later time points. However, we have shown that a PMAxx^TM^-based qPCR scheme is useful for estimating viable *S.* Infantis caecal load, the assay proved to be efficient, sensitive, and repeatable. Certainly, this approach could be utilized in studies comparing the effects of diverse treatments on *Salmonella* caecal content. 

## Figures and Tables

**Table 1:** Reproducibility of the real-time PCR method for the *invA* gene.

Dilution	Ct1x	Ct2x	Ct3x	Average Ct	SD	CV (%)
−1	16.93	16.94	17.07	16.98	0.06	0.38
−2	20.19	20.49	20.88	20.52	0.28	1.38
−3	23.97	24.26	24.25	24.16	0.13	1.54
−4	28.83	28.81	28.88	28.84	0.03	0.10
−5	32.56	32.46	32.62	32.55	0.07	0.21

SD, standard deviation; CV, coefficient of variation (*n* = 3).

## Data Availability

Data available as Supplementary Information.

## Supplementary Materials

The following supporting information can be downloaded at: https://www.mdpi.com/article/10.3390/vetsci9090487/s1, Table S1: Components of the qPCR reaction; Table S2: COBB 500 Feed components and proximate composition of starter (0–8 days) diets; Figure S1: DNA integrity; Supplementary Data.
