# Supplementary material for: A PMAxxTM qPCR Assay Reveals That Dietary Administration of the Microalgae Tetraselmis chuii Does Not Affect Salmonella Infantis Caecal Content in Early-Treated Broiler Chickens
Source: Vet Sci. 2022 Sep 8;9(9):487. doi: 10.3390/vetsci9090487 (PMC9503589; doi:10.3390/vetsci9090487)
Supplement: Supplementary file 1 [file vetsci-09-00487-s001.zip › Figure S1.pdf]

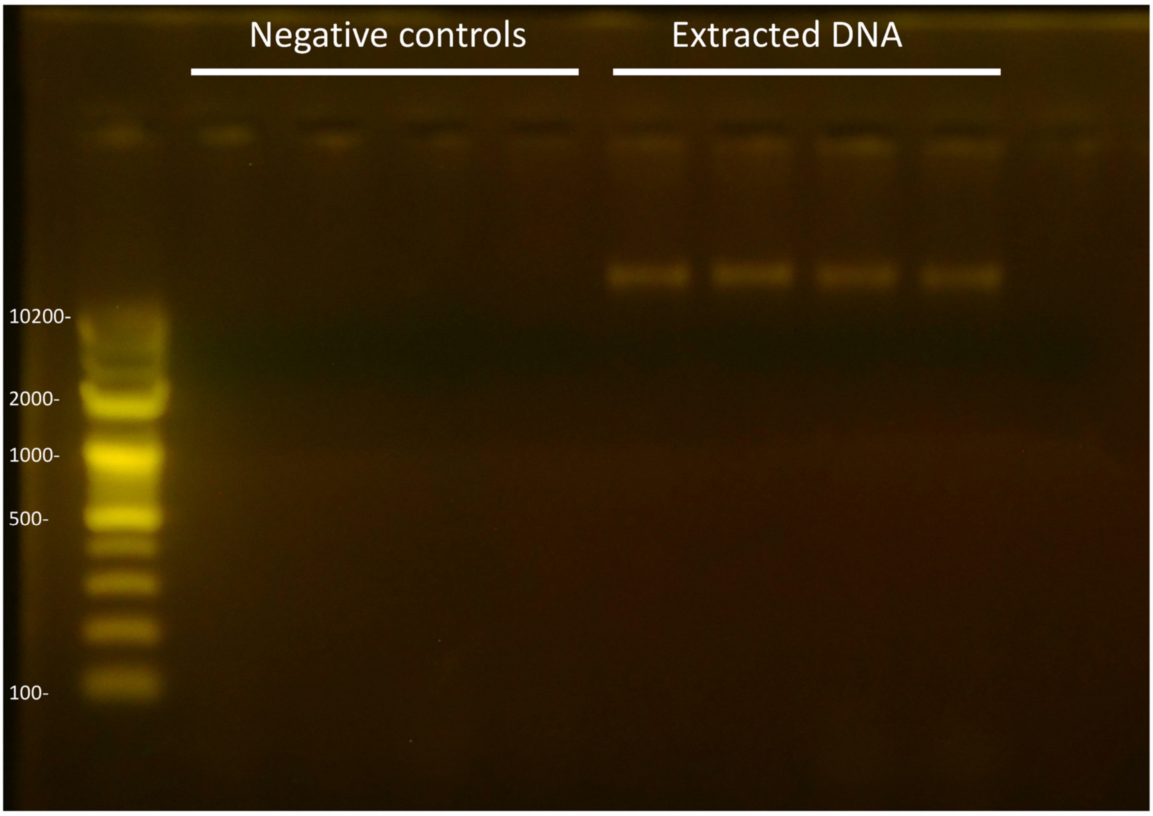

Figure S1. DNA integrity observation by electrophoresis in agarose gel. DNA samples were analyzed on a 0.8% agarose gel. The 100 bp Plus DNA Ladder (MW) was also separated as size reference.
