# Supplementary material for: A PMAxxTM qPCR Assay Reveals That Dietary Administration of the Microalgae Tetraselmis chuii Does Not Affect Salmonella Infantis Caecal Content in Early-Treated Broiler Chickens
Source: Vet Sci. 2022 Sep 8;9(9):487. doi: 10.3390/vetsci9090487 (PMC9503589; doi:10.3390/vetsci9090487)
Supplement: Supplementary file 1 [file vetsci-09-00487-s001.zip › Table S1 and Table S2.pdf]

Table S1. Components of the qPCR reaction.		
**Reaction components**	**Amount per 20 µL of reaction (µL)**	**Final concentration**
2X Forget-Me-Not Master Mix	10	1X
invA primer mix	2	0.5 µm
DNA template	5	X
High ROX marker	0.5 µL (optional)	–
dH_2O	3	–

Table S2. COBB 500 Feed components and proximate composition of starter (0–8 days) diets		
**Ingredients (%)**	Control	Experimental group
Ground corn	52.99	52.88
Soybean meal	36.3	36.23
Calcium carbonate	1.52	1.52
Monocalcium phosphate	1.07	1.07
Sodium chloride	0.31	0.31
Crude (vegetal) Fat	7.4	7.39
Antimycotic	0.10	0.10
Mycotoxin Sequestrant	0.05	0.05
Antioxidant	0.02	0.02
Phytase	0.01	0.01
[1]Vitamin and mineral premix	0.23	0.23
Microalgae-derived protein	-	0.08
Microalgae-derived fat	-	0.01
Microalgae-derived crude ash	-	0.05
Microalgae-derived carbohydrate, fiber, rest of biomass	-	0.06
**Nutrient specifications**		
[2]ME Kcal/kg diet	2975	3025
Digestible Lysine (%)	1.22	1.12
Digestible Methionine + Cysteine (%)	0.91	0.85
Digestible Methionine (%)	0.46	0.45
Digestible Threonine (%)	0.83	0.73
Digestible Valine (%)	0.89	0.85
Digestible Isoleucine (%)	0.77	0.72
Digestible Arginine (%)	1.28	1.18
Digestible Tryptophan (%)	0.20	0.18
Crude protein (%)	21.50	20.00
Ca (%)	0.90	0.84
Available P (%)	0.45	0.42
Na (%)	0.23	0.16
Cl (%)	0.22	0.19
K (%)	0.95	0.72
Choline (mg/kg)	500	400
Linoleic acid (%)	1.00	1.00

[1]Vitamin premix incorporated in each kg of basal diets: Vitamin A 10,000 IU; vitamin D3 5000 IU; vitamin E 80 IU; vitamin K 3 mg; vitamin B1 3 mg; vitamin B2 9 mg; vitamin B6 4 mg; vitamin B12 0.02 mg; Biotin 0.15 mg; Pantothenic acid 15 mg; Folic acid 2 mg. Mineral premix incorporated in each kg of basal diets: Mn, 100 mg; Zn, 100 mg; Fe, 40 mg, Cu, 15 mg, I, 1 mg, Se, 0.35 mg. [2]ME, metabolizable energy.
